# Bilateral Adrenal Hemorrhage After Laminectomy: A Rare Complication

**DOI:** 10.1210/jcemcr/luaf328

**Published:** 2026-02-04

**Authors:** Sapna Sharma, Michelle Ashley Rizk, Hafiza Qadeer, Rubina Paudel, Chheki Sherpa

**Affiliations:** Department of Endocrinology, Diabetes and Metabolism, Reading Hospital, Reading, PA 19610, USA; Department of Endocrinology, Diabetes and Metabolism, Reading Hospital, Reading, PA 19610, USA; Department of Endocrinology, Diabetes and Metabolism, Reading Hospital, Reading, PA 19610, USA; Department of Endocrinology, Diabetes and Metabolism, Reading Hospital, Reading, PA 19610, USA; Department of Endocrinology, Diabetes and Metabolism, Reading Hospital, Reading, PA 19610, USA

**Keywords:** adrenal insufficiency, laminectomy, bilateral adrenal hemorrhage, postoperative adrenal hemorrhage, endocrine emergency, adrenal crisis

## Abstract

Bilateral adrenal hemorrhage is a rare but life-threatening condition that can result in adrenal insufficiency. We present the case of a 63-year-old man who developed bilateral adrenal hemorrhage following a fall from 3 feet height on a ladder. He fell backwards, striking his lower back on the adjacent dry wall and landed on the ground. He was admitted for a traumatic L1 burst fracture and was treated with T11-L3 fusion and T12-L3 laminectomy. Postoperatively, he received prophylactic subcutaneous unfractionated heparin and later developed unexplained tachycardia, pulmonary embolism, and bilateral adrenal masses. He was subsequently readmitted with altered mental status, hypotension, and profound electrolyte abnormalities. Laboratory evaluation revealed undetectable cortisol and elevated adrenocorticotropic hormone (ACTH), consistent with primary adrenal insufficiency. Imaging confirmed hyperdense bilateral adrenal masses. Infectious causes were excluded, and heparin-induced thrombocytopenia was ruled out. The patient was treated with intravenous hydrocortisone, leading to rapid clinical improvement. He was discharged on oral steroid therapy and remains well on follow-up. This case highlights the importance of considering bilateral adrenal hemorrhage in postoperative patients presenting with nonspecific symptoms and hemodynamic instability.

## Introduction

Laminectomy is a commonly performed surgical procedure for various spinal conditions. While it is considered safe, rare complications can occur. Bilateral adrenal hemorrhage is an exceedingly uncommon complication of spinal surgery. We describe a case of bilateral adrenal hemorrhage following laminectomy and discuss the diagnostic and management strategies. In patients with any trauma, adrenal neoplasm, surgeries, or procedures who present with unexplained symptoms of abdominal pain, back pain, hypotension, fever, confusion, or electrolyte abnormalities, especially hyponatremia, acute adrenal insufficiency should be highly suspected [1].

## Case Presentation

**Figure 1.**
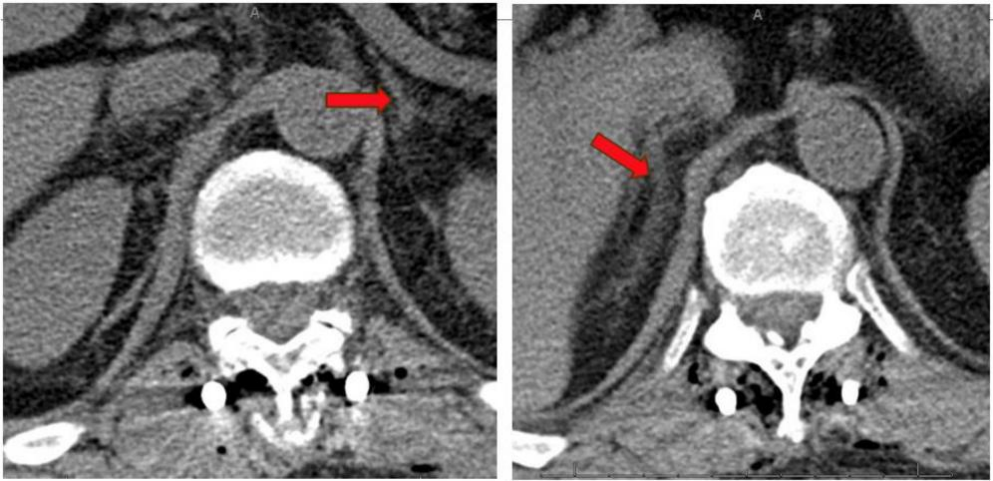
CT of thoracic/lumbar spine revealing normal-appearing left and right adrenal glands after the fall.

**Figure 2.**
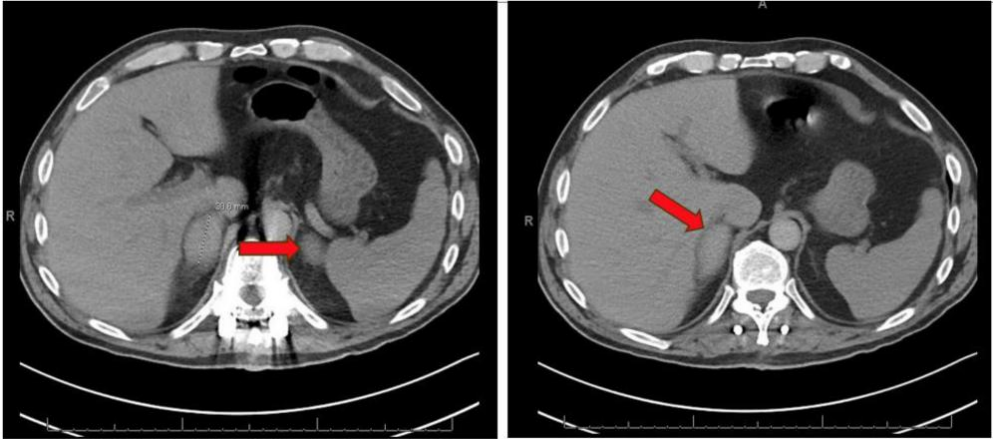
CT angiogram of the chest (pulmonary embolism protocol) revealing hyper-enhancing bilateral enlargement of the adrenal glands post-procedure before the diagnosis of primary adrenal insufficiency.

**Figure 3.**
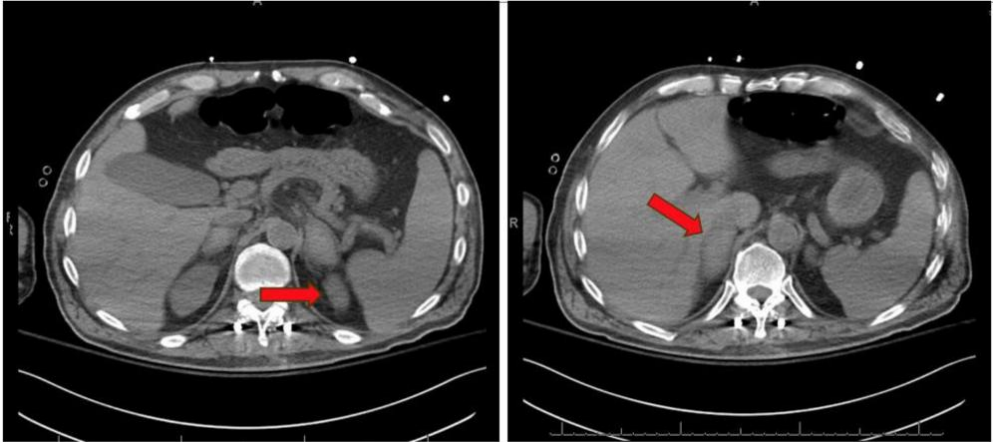
Timeline of events leading to diagnosis.

A 63-year-old man with past medical history of hypertriglyceridemia presented to the hospital after a 3-foot fall from a 6-foot ladder. He fell backwards, striking his lower back on the adjacent dry wall and landed on the ground. He was admitted for a traumatic L1 burst fracture and was treated with T11-L3 fusion and T12-L3 laminectomy. He was subsequently started on prophylaxis for venous thromboembolism with unfractionated heparin 5000 units subcutaneously every 8 hours. The patient had normal bilateral adrenal glands after the fall as shown on computed tomography (CT) thoracic/lumbar spine imaging (see Fig. 1). He was discharged to acute rehabilitation. One week later, while at acute rehabilitation he developed fever, tachycardia, and elevated white blood cell count 13.4 × 10^3^/μL (SI: 13.4 10^9^/L; reference: 4.8-10.8 × 10^3^/μL [4.8-10.8 × 10^9^/L]), evaluation including duplex ultrasound was negative for deep vein thrombosis and CT angiogram was also negative for pulmonary embolism but was concerning for pneumonia and was treated with antibiotics. The course was also complicated by moderate thrombocytopenia (platelet count 57 × 10^3^/μL [SI: 57 × 10^9^/L]; reference 130-400 × 10^3^/μL [SI: 150-400 × 10^9^/L]), initially thought to be induced by prophylactic heparin since heparin antibody was positive. He was transitioned to fondaparinux for prophylaxis. Serotonin release assay was sent and returned negative weeks later ruling out heparin-induced thrombocytopenia (HIT). Two weeks later, he developed unexplained tachycardia and hence CT chest angiogram was repeated, which showed segmental and subsegmental pulmonary embolism. The CT angiogram also demonstrated bilateral adrenal nodules measuring at least 3.9 cm on right side and 3.3 cm on left side (see Fig. 2), but a dedicated adrenal imaging was not done during that admission. The patient was treated with therapeutic fondaparinux and was discharged home on apixaban 10 mg 2 times daily. Three weeks later (see Fig. 3), he was readmitted after his wife reported poor appetite, weight loss of 30 lbs, weakness, and altered mental status. No fever, cough, congestion, diarrhea, abdominal pain, dysuria, or fall were reported at that time. He was lethargic and awake but unable to follow commands.

## Diagnostic Assessment

**Figure 4.**
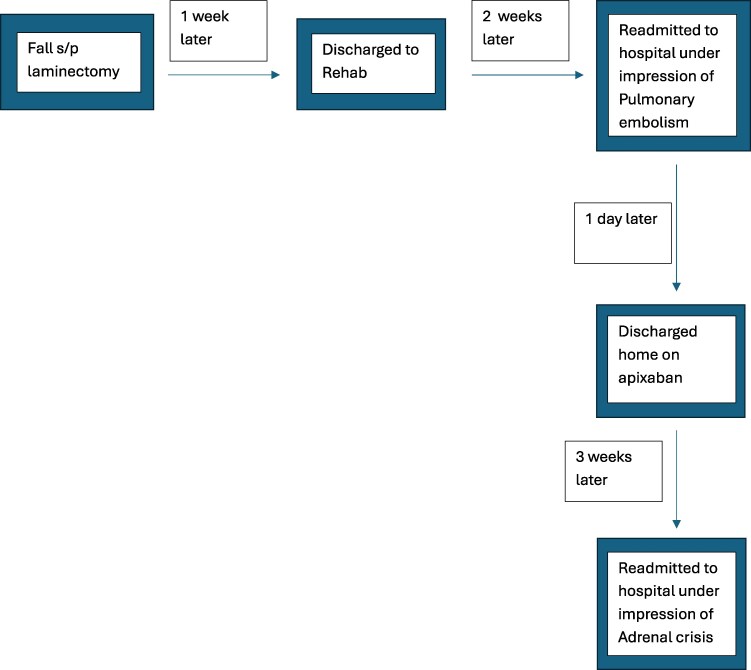
CT of the chest, abdomen, and pelvis without intravenous contrast revealing worsening enlargement of bilateral adrenal glands at the time of diagnosis of primary adrenal insufficiency.

His vital signs on admission were significant for hypotension (91/44 mmHg) and tachycardia (116 bpm). Initial workup showed profound hyponatremia with sodium 121 mEq/L (SI: 121 mmol/L) (reference 136-145 mEq/L [SI: 135-145 mmol/L]), hyperkalemia with potassium 6.1 mmol/L; reference 3.5-5.3 mmol/L), hypoglycemia with glucose 58 mg/dL (SI: 3.22 mmol/L) (reference 70-99 mg/dL [SI: 3.9-5.5 mmol/L]), hypercalcemia showing calcium of 11.7 mg/dL (SI: 0.65 mmol/L) (reference 8.6-10.3 mg/dL [SI: 2.18-2.58 mmol/L]), and acute kidney injury with creatinine 2.58 mg/dL (SI: 228 µmol/L; reference 0.6-1.3 mg/dL [53-114.9 µmol/L]). He received initial resuscitative measures for hypovolemia and electrolyte abnormalities. Morning serum cortisol was noted to be undetectable (<1.0 μg/dL) (normal: 6.7-22.6 μg/dL) and adrenocorticotropin hormone (ACTH) level, which resulted later, was high at 329 pg/mL (72.33 pmol/L) (reference 6-50 pg/mL [1.3-16.7 pmol/L]). The aldosterone level was normal 3 ng/dL (83.22 pmol/L; reference 3-16 ng/dL [83.22-832.2 pmol/L]) and renin levels were elevated to 22.9 ng/mL/h (SI: 22.9 μg/L/h; reference: 0.25-5.82 ng/mL/h [0.2-4.3 μg/L/h]). The patient was started on stress-dose steroids for suspected adrenal crisis. Repeat CT scan of chest/abdomen/pelvis without contrast was done to further characterize adrenal nodules, since initial imaging was done with contrast which showed bilateral hyperdense 4 cm adrenal mass/hematoma (Fig. 4). The initial CT of thorax/spine done after the fall showed normal adrenal glands and hence the new bilateral hyperdense adrenal masses noted after the procedure were suggestive of adrenal hemorrhage/hematoma.

Probable causes considered for bilateral adrenal hemorrhage were anticoagulant use, HIT, and infectious causes, including Waterhouse-Friderichsen syndrome (meningococcemia-related bilateral adrenal hemorrhage). Infectious workup, including blood and urine cultures, was negative. A lumbar puncture was deferred due to improvement of the mental status with steroids. HIT was ruled out since serotonin release assay was negative.

## Treatment

The patient was immediately started on intravenous hydrocortisone 100 mg followed by 50 mg every 6 hours followed by slow taper. He responded well to glucocorticoid replacement with improvement of electrolyte abnormalities and mental status. He also received treatment for community acquired pneumonia with broad spectrum antibiotics. He was discharged to acute rehabilitation on oral glucocorticoid therapy.

## Outcome and Follow-up

Currently the patient is following with the endocrinology outpatient clinic. He is maintained on oral hydrocortisone 15 mg in the morning and 5 mg in the evening plus fludrocortisone 0.1 mg daily. His fatigue has improved, he is gaining weight, and electrolytes are normal. He is now back at work.

## Discussion

Bilateral adrenal hemorrhage leading to adrenal insufficiency can cause significant morbidity and mortality if not diagnosed promptly [2]. Bilateral adrenal hemorrhage in a postoperative state is a rare condition. Bilateral adrenal hemorrhage in the setting of heparin-induced thrombocytopenia has been reported after multiple surgical procedures, such as hip arthroplasty, total knee arthroplasty, and coronary artery bypass grafting [3-7]. To our knowledge, this is the first case report to describe bilateral adrenal hemorrhage following laminectomy secondary to predisposing factors discussed below.

Bilateral adrenal hemorrhage after surgery most commonly occurs due to a combination of severe physiological stress and predisposing factors such as coagulopathy, sepsis, or exposure to anticoagulants [8]. Our patient had thrombocytopenia post-laminectomy, and it was thought to be due to HIT. However, his serotonin release assay came back negative, ruling out HIT in this case. The unique blood supply of adrenal gland has been hypothesized to be a possible mechanism which puts it at risk of thrombosis and hemorrhage. Each adrenal gland has 3 arterial feeding vessels with a network of nearly 60 arterioles. However, it is drained by a single central adrenal vein which leads to an abrupt transition of blood flow, making the gland vulnerable to hemorrhage [1, 2, 9-11]. Stress and subsequent rise in catecholamines can increase adrenal blood flow and venous stasis, leading to vascular congestion and makes it vulnerable leading to hemorrhage [10, 12]. Surgical procedures impose significant physiological stress, triggering a substantial release of catecholamines and ACTH. The elevated concentrations of these hormones within the adrenal vein contribute to venoconstriction and enhanced platelet aggregation, potentially leading to adrenal vein thrombosis [13]. Postmortem studies have demonstrated venous thrombosis within the adrenal glands of individuals with bilateral adrenal hemorrhage. This phenomenon is hypothesized to result from an excessive release of catecholamines, thrombin, fibrin, and endotoxins, which collectively elevate adrenal vascular pressure and precipitate hemorrhage accompanied by injury to the adrenal medulla [14, 15]. Adrenal glands have a vulnerable single-vein outflow system, making them more susceptible to congestion and hemorrhage after surgery, unlike the vertebral vasculature with more collateral flow [13]. Interestingly, the 11th and 12th thoracic vertebrae are supplied by the posterior intercostal arteries which branch from the thoracic aorta. The middle suprarenal arteries branch directly from the abdominal aorta, and the adrenal glands are located at the same level as that of the 11th and 12th thoracic vertebrae. The superior suprarenal arteries arise from the inferior phrenic artery which is also a branch from abdominal aorta. In contrast, the inferior suprarenal arteries branch directly from the renal arteries although variations can exist [16].

Bilateral adrenal hemorrhage leading to adrenal insufficiency presents as nonspecific symptoms like abdominal pain, nausea, vomiting, fever, fatigue, and dizziness and clinical signs like hypotension unresponsive to fluids and vasopressors, fever, and tachycardia [1, 3]. These signs and symptoms associated with sudden hyponatremia, hyperkalemia, and hypoglycemia increase suspicion of acute adrenal insufficiency. In this case, our patient had a recent laminectomy, and he presented with symptoms of generalized weakness, weight loss, hypotension, and electrolyte abnormalities such as hyponatremia, hypoglycemia, hyperkalemia, and hypercalcemia. Patients with adrenal hemorrhage experience electrolyte imbalances, which may involve hyponatremia, hyperkalemia, hypoglycemia, and hypercalcemia [9]. In this case, primary adrenal insufficiency was suspected and was confirmed with undetectable Am cortisol and an elevated ACTH level of 329 pg/mL.

Anatomical diagnosis of adrenal hemorrhage can be made with abdominal CT scan or magnetic resonance imaging (MRI) showing uniform, rounded enlargement of adrenal glands with homogenous enhancement [3]. Non-contrast abdominal CT is sufficient and thought to be standard diagnostic assessment in most acutely ill patients but sometimes it is difficult to interpret especially when CT is performed in acute hemorrhagic phase. Our patient had an initial CT angiography with contrast to rule out pulmonary embolism, which did not show adrenal hemorrhage. It is important to note that a dedicated adrenal imaging with CT would not have altered management of adrenal hemorrhage. MRI adrenals have the advantage of high accuracy and are the most sensitive and specific imaging tool to differentiate between adrenal hematoma from adjacent necrotic tissue and determining time of hematoma [10, 11].

It is notable that adrenal insufficiency secondary to bilateral adrenal hemorrhage has an insidious presentation and adrenal crisis develops in the setting of stress. Likewise, our patient had developed adrenal hemorrhage, evidenced by the presence of bilateral adrenal gland lesions postoperatively in CT scan.

Because of life-threatening complications, bilateral adrenal hemorrhage–associated adrenal insufficiency should be diagnosed promptly and managed to prevent death [3, 17]. As early diagnosis and prompt treatment can decrease mortality rate, empiric treatment is recommended before confirming diagnosis, if there is a high suspicion of adrenal insufficiency. Acute management of adrenal insufficiency is the same irrespective of etiology [2, 3]. Glucocorticoid replacement is the mainstay of treatment [17]. Intravenous hydrocortisone with 100 mg bolus then 200 mg per day in divided doses along with fluid resuscitation is recommended in acute phase followed by steroid taper with clinical improvement [2, 3]. For long-term treatment of bilateral adrenal hemorrhage–induced adrenal insufficiency, maintenance dosing of hydrocortisone and fludrocortisone is recommended. Intermittent assessment of adrenal function can be done to look for recovery. Recovery of adrenal function after sustaining bilateral hemorrhage is not well known, but the damage seems to be permanent in many cases, with only 6% resolution of primary adrenal insufficiency [12]. This case report emphasizes the significance of timely diagnosis and management of bilateral adrenal hemorrhage, which leads to primary adrenal insufficiency. Bilateral adrenal hemorrhage is an exceptionally rare complication following laminectomy. The likely multifactorial etiology of bilateral adrenal hemorrhage in this case involves initial microvascular adrenal injury from trauma, which was further exacerbated by surgical stress. Additional contributing factors included transient thrombocytopenia, perioperative use of heparin, fondaparinux, and apixaban collectively increasing the risk of hemorrhage. Clinicians should be aware of this possibility and consider it in the differential diagnosis of postoperative abdominal pain and electrolyte abnormalities, especially hyponatremia.

## Learning Points

- Bilateral adrenal hemorrhage should be considered in postoperative patients presenting with nonspecific symptoms such as hypotension, tachycardia, altered mental status, and electrolyte abnormalities.
- Adrenal insufficiency secondary to bilateral adrenal hemorrhage can mimic sepsis or other critical illnesses, making it essential for clinicians to maintain a high index of suspicion in patients with unexplained clinical deterioration, especially in the postoperative setting.
- Prompt recognition and treatment of adrenal insufficiency with stress-dose corticosteroids is lifesaving and should be initiated without delay when clinical and laboratory findings suggest adrenal crisis, even before confirmatory testing is available.

## Contributors

All authors made individual contributions to authorship. M.R., R.P., and C.S. were involved in the diagnosis and management of the patient and S.S., H.Q., and C.S. were involved in acquisition and interpretation of data and drafting of case report. All authors reviewed and approved the final draft.

## Data Availability

Data sharing is not applicable to this article as no datasets were generated or analyzed during the current study.

## Abbreviations

ACTH: adrenocorticotropin
CT: computed tomography
HIT: heparin-induced thrombocytopenia
MRI: magnetic resonance imaging
